# Repositioning Dequalinium as Potent Muscarinic Allosteric Ligand by Combining Virtual Screening Campaigns and Experimental Binding Assays

**DOI:** 10.3390/ijms21175961

**Published:** 2020-08-19

**Authors:** Angelica Mazzolari, Silvia Gervasoni, Alessandro Pedretti, Laura Fumagalli, Rosanna Matucci, Giulio Vistoli

**Affiliations:** 1 Dipartimento di Scienze Farmaceutiche, Università degli Studi di Milano, Via Mangiagalli, 25, I-20133 Milano, Italy; angelica.mazzolari@unimi.it (A.M.); silvia.gervasoni@unimi.it (S.G.); alessandro.pedretti@unimi.it (A.P.); laura.fumagalli@unimi.it (L.F.); 2 Dipartimento di Neuroscienze, Psicologia, Area del Farmaco e Salute del Bambino (NEUROFARBA), Sezione di Farmacologia e Tossicologia, Università degli Studi di Firenze, Viale Pieraccini 6, 50139 Firenze, Italy; rosanna.matucci@unifi.it

**Keywords:** drug repurposing, virtual screening, consensus function, binding space, muscarinic receptors, allosteric modulators

## Abstract

Structure-based virtual screening is a truly productive repurposing approach provided that reliable target structures are available. Recent progresses in the structural resolution of the G-Protein Coupled Receptors (GPCRs) render these targets amenable for structure-based repurposing studies. Hence, the present study describes structure-based virtual screening campaigns with a view to repurposing known drugs as potential allosteric (and/or orthosteric) ligands for the hM_2_ muscarinic subtype which was indeed resolved in complex with an allosteric modulator thus allowing a precise identification of this binding cavity. First, a docking protocol was developed and optimized based on binding space concept and enrichment factor optimization algorithm (EFO) consensus approach by using a purposely collected database including known allosteric modulators. The so-developed consensus models were then utilized to virtually screen the DrugBank database. Based on the computational results, six promising molecules were selected and experimentally tested and four of them revealed interesting affinity data; in particular, dequalinium showed a very impressive allosteric modulation for hM_2_. Based on these results, a second campaign was focused on bis-cationic derivatives and allowed the identification of other two relevant hM_2_ ligands. Overall, the study enhances the understanding of the factors governing the hM_2_ allosteric modulation emphasizing the key role of ligand flexibility as well as of arrangement and delocalization of the positively charged moieties.

## 1. Introduction

Drug repositioning represents an efficient strategy to find novel therapeutic applications for old molecules [1,2,3,4]. The relevance of such a strategy is clearly understandable when considering that unsuitable pharmacokinetic profiles represent a critical issue in drug attrition and the failures due to toxicological outcomes are even increasing in the last years [5]. While considering the significant efforts which are now invested in early toxicological screenings, it is no doubt that a discovery project based on an old drug—whose toxicity and safety have been already extensively investigated in humans—can resolve ab initio all related problems. Moreover, the polypharmacology paradigm has afforded a conceptual validation to drug repositioning studies [6] suggesting that the ability of a given molecule to bind diverse targets may increase its therapeutic efficacy especially when treating complex diseases [7,8].

Among the amenable strategies for drug repositioning studies, docking-based virtual screening (VS) represents a very efficient method provided that reliable 3D structures of the involved protein targets are available [9]. Thanks to the recent advancements in their resolution, GPCRs are becoming suitable targets for VS analyses [10] and, among them, human muscarinic receptors (hmAChRs) are of particular relevance when considering that all the five subtypes have been resolved including both active and inactive conformations [11,12,13]. The hM_2_ subtype is particularly suitable for structure-based analyses since it has been resolved in its inactive state in complex with an antagonist [14] as well as in its active state in complex with both an agonist and an allosteric modulator [15].

This last hM2 crystal structure is particularly useful for VS campaigns aimed to identify novel allosteric ligands since it allows a precise definition of the architecture of the allosteric binding cavity. The general interest for allosteric modulators has exploded in the recent years since they can enhance the ligand’s subtype-selectivity especially when it can be reached with difficulty by simple orthosteric ligands [16]. Notably, this problem is particularly exacerbated for muscarinic receptors where the highly conservation degree of the key residues lining the orthosteric cavity prevents the design of truly selective ligands [17].

**Figure 1:**
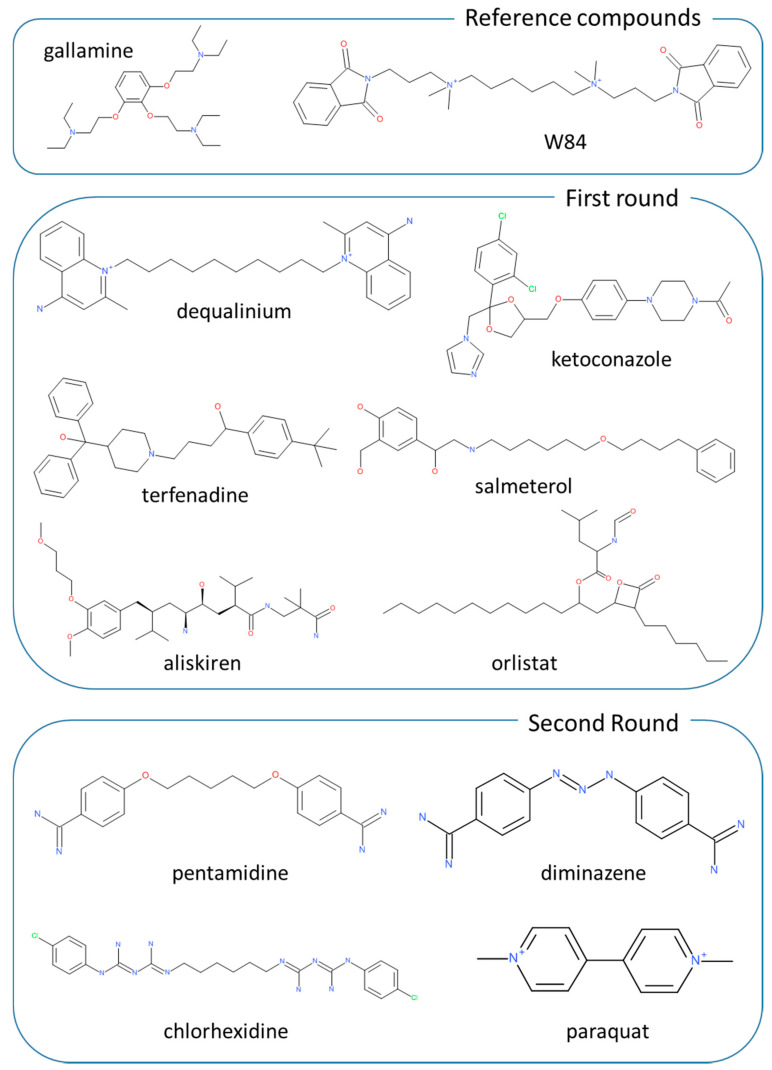
Chemical structures of the retrieved hits from virtual screening (VS) campaigns which were experimentally investigated by binding assays. The chemical structures of the two reference compounds (namely, gallamine and W84) were also included.

On these grounds, a VS study on the hM_2_ subtype was undertaken with a view to finding novel allosteric modulators by screening an extended dataset of known drugs. The study was organized in three steps. In the first preliminary part, an efficient VS strategy was developed by considering and combining two representative resolved hM_2_ structures and a purposely collected database including 30 known allosteric modulators which were dispersed among 2970 suitably selected decoys. As already validated in previous analyses [18,19], the VS strategy involved an extensive rescoring of the computed docking results combined with the development of linear consensus predictive models by applying the enrichment factor optimization algorithm (EFO) [20]. Then, the so tuned in silico procedures were utilized to screen a DrugBank-based database [21] including about 6000 known drugs and the resulting six most promising ligands (see Figure 1) were experimentally tested. Given the remarkable experimental results afforded by dequalinium, the third step involved the collection of a focused small set of commercially available bis cationic derivatives, which were similarly analyzed and experimentally tested to assess their biologic activity on hM_2_ and to investigate the corresponding structure–activity relationships.

## 2. Results

### 2.1. Preliminary Virtual Screening Simulations

Previous comparative studies emphasized the efficacy of the EFO approach to develop consensus models by linearly combining diverse scoring functions and/or different docking engines [18,19]. The obtained results revealed that EFO-based equations including three docking scores are able to provide enhanced predictive powers outperforming most of the available consensus and scoring strategies. On the other hand, previous docking studies highlighted that the binding space concept [22], which combines more than one computed pose for each ligand, proves successful in accounting for the multiple binding modes a ligand assumes within the binding cavity. In detail, previous studies suggested that average and range values are suitable descriptors to explore such a binding space and can find fruitful applications for developing predictive models.

Notwithstanding these encouraging results, the application of the binding space descriptors to optimize the performances of virtual screening campaigns was never investigated because docking simulations for virtual screening usually generate only one pose per ligand, a choice clearly explainable for minimizing computational costs. On these grounds, these preliminary simulations were carried out also to investigate the beneficial role of considering 10 poses per ligand as encoded by the binding space descriptors when developing EFO-based consensus linear equations. Moreover, the here reported application allows an extended investigation of the binding space effects by combining 10 poses per ligand as well as two different hM_2_ conformations.

Indeed, as mentioned in the Introduction, this study was focused on the hM_2_ receptor due to the availability of two relevant resolved structures: the open inactive state in complex with quinuclidinyl benzilate (QNB, PDB Id: 3UON) and the close active state in complex with both an agonist (iperoxo) and an allosteric modulator (LY211962, PDB Id: 4MQT). As reported by Kruse et al. [15], the comparison of the two structures reveals that the orthosteric site of the active state is smaller than the inactive one and indeed is unable to accommodate QNB. With regard to the allosteric pocket, the vestibule above the orthosteric site is visible in both structures even though the narrowness characterizing the active state is also reflected in the allosteric site which appears to be smaller due to the rotation of TM6. The comparison with the resolved active state in complex with the iperoxo agonist—but without LY211962 (PDB Id: 4MQS)—reveals that the overall architecture of the allosteric site is conserved even without the allosteric ligand and only one residue (i.e., Trp422) appears differently arranged in the ternary complex reasonably to optimize the interactions with the allosteric modulator. For these reasons, the here performed simulations involved the active state in ternary complex which should have the allosteric pocket perfectly suited to accommodate its ligands, and the open inactive state in order to investigate its capacity to harbor larger modulators. In contrast, the resolved active state in complex with the sole agonist was not considered since this should afford docking results almost identical to those provided by the ternary active complex.

Table 1 compares the performances, as encoded by the corresponding enrichment factor (EF) 1% values, reached by the here performed VS simulations when applying the binding space concept or focusing on the best pose only. For a better comparison, Table 1 reports both the highest EF 1% values and the EF 1% means as computed by averaging the performances of all the best 10 generated models. The first consideration involves the different performances between the two hM_2_ structures since docking results generated by using 4MQT perform clearly better in all experiments. Regardless of the conformational differences between the two hM_2_ states, such a result can be explained by considering that 4MQT was resolved in complex with an allosteric modulator and so its allosteric cavity should be well tuned to accommodate allosteric ligands. In contrast, 3UON is in its inactive and open state and was resolved without allosteric modulators. As discussed above, its allosteric pocket is not fully suited to harbor these ligands and may be wider than that required for a convenient recognition.

The observed differences between the two hM_2_ structures are reflected into the effects of binding space parameters. Indeed, the performances reached by using the poses as computed for the inactive state (PDB Id: 3UON) remarkably benefit from multiple poses, as evidenced by the notably better enrichment factors that score averages provide compared to the best score only. In contrast, docking results generated by using the active state (PDB Id: 4MQT) afford comparable EF 1% results when considering either the best scores or the average values. The different impact of the score averages suggests that the optimal conformation of the 4MQT allosteric pocket reduces the mobility of the docked ligands constraining them to assume an almost unique (and reasonably correct) binding mode which is conveniently parameterized even by using the best docking score only. In contrast, the wider cavity of the 3UON structure permits a greater mobility to the docked ligands which can consequently assume different binding modes. The best score does not always correspond to the correct pose, as evidenced by the less satisfactory performances provided by the best scores only, thus score averages appear to be a convenient way to account for the correct binding modes. This is clearly appreciable by considering that multiple poses afford a doubled best EF 1% value.

Interestingly, the inclusion of the range values affords comparable and not negligible improvements for the docking results produced by both hM_2_ structures. This result emphasizes that the range values encode for different information compared to the score averages since they presumably describe the mobility a given ligand retains during binding and thus encode for ligand flexibility/entropy, the inclusion of which shows beneficial effects regardless of the reliability of the protein structure. Unfortunately, the combination of the docking results from the two simulated proteins does not produce additional enhancements, an unsatisfactory result which can be explained by considering the different performances exhibited by docking results of the two hM_2_ structures. Indeed, their combination affords results always comparable to those obtained by using 4MQT only.

Table 2 compiles the best models generated by the EFO approach for each experiment. As detailed under Methods, the consensus equations are generated by linearly combining the computed binding space parameters and including in each equation at most three variables. The equations developed by combining the docking results of both proteins (3UON + 4MQT, see Table 1) were omitted since they roughly correspond to those produced by using the results from 4MQT only. A bird’s eye analysis of the included variables reveals a massive occurrence of the PLANTS scores to confirm their capacity to suitably describe the simulated binding process. The scores are often included as normalized values which should minimize the effect of the unavoidable differences in molecular size among the screened ligands. Notably, both equations including average and range values comprise two score averages and one range value which may encode for the entropic factors as discussed above.

To summarize these preliminary docking simulations, one may conclude that: (1) the utilized docking strategies should prove successful in recognizing potential allosteric modulators in the future VS campaigns; (2) averaging docking scores from multiple binding poses appears particularly useful when using non-optimal protein structures; (3) the range values induce limited, but constant enhancements presumably since they encode for the often disregarded entropic factors. Taken together, the obtained results confirm the remarkable potentials that the binding space concept can also have when analyzing VS simulations and invite to a more extended validation to assess its beneficial role.

**Figure 2:**
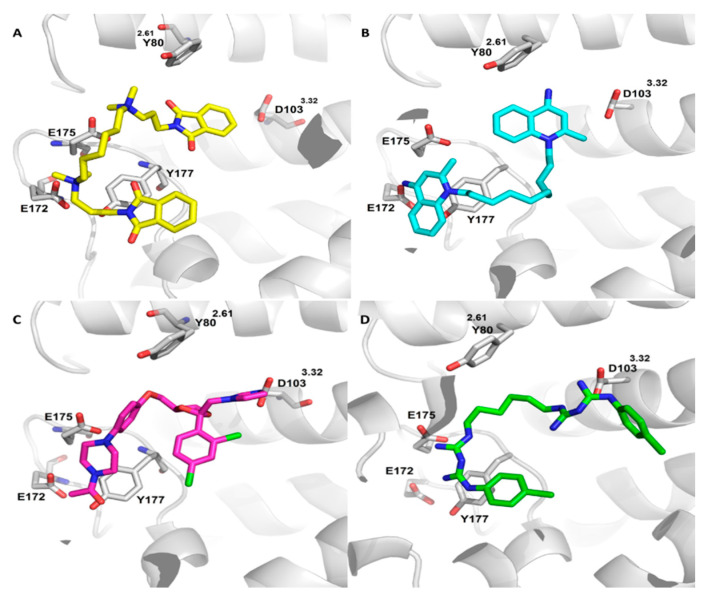
Putative complexes as computed by docking simulations for (yellow carbon atoms, (**A**) W84, (azure carbon atoms, (**B**) dequalinium, (purple carbon atoms, (**C**) ketoconazole and (green carbon atoms, (**D**) chlorhexidine within the hM_2_ binding sites in its active state (PDB Id: 4MQT). In all figures, the loop between residues 411 and 418 was not displayed for sake of clarity.

To give a glimpse of the major interactions characterizing the allosteric binding pocket, Figure 2A shows the putative complex as computed for the known and potent W84 allosteric modulator during these preliminary docking simulations. The ligand assumes a rather folded pose by which it completely occupies the allosteric pocket. In detail, the ammonium heads are engaged in key ion pairs with Glu172 and Glu175 reinforced by charge transfer interactions involving surrounding aromatic residues (i.e., Tyr80 and Tyr177). The two phthalimido moieties elicit similar interaction patterns comprising π–π stacking interactions plus H-bonds. Interestingly, here and in most generated poses, W84 inserts a phthalimido ring within the orthosteric site without eliciting the required ion-pair with Asp103. These complexes are in agreement with the binding parameters determined for W84 (see Table 3) emphasizing that W84, while showing an intermediate pK_i_ value, primarily acts as allosteric modulator as evidenced by its high log K_occ_ value.

### 2.2. Virtual Screening for Drug Repositioning

As mentioned under Methods and based on the results of preliminary VS analyses, docking simulations on DrugBank database were focused on the 4MQT structure only. The analysis of the obtained results was based on a sort of consensus of the consensus models since the computed docking scores were utilized by including them into all the three highly performing equations based on the 4MQT structure (see Table 2). Hence, each equation was utilized to generate a ranking including all DrugBank molecules and the final overall consensus was computed by averaging the position of each ligand in the three corresponding rankings. Attention was paid on the first 100 molecules from which 30 marketed drugs (Table 3) were extracted, avoiding experimental molecules and compounds which are under development phases and thus are not easily available. A qualitative analysis of the selected compounds reveals two major groups of molecules. The first group is composed by cationic compounds of different lipophilicity and comprises aminergic ligands (such as salmeterol or terfenadine), cholinesterase inhibitors (demecariun or ambenonium) and disinfectants (such as dequalinium). The second group comprises highly lipophilic drugs (e.g., orlistat and ketokonazole) and compounds related to prostaglandins (such as iloprost).

By considering the obtained top ranked ligands, the tested molecules were selected based on these criteria: for the first group, priority was given to the lipophilic cationic molecules, avoiding metabolically labile compounds, while for the second group priority was given to neutral molecules. As shown in Figure 1, from the first group, terfenadine, salmeterol and aliskiren were chosen as cationic compounds as well as dequalinium as representative of a bis-cationic derivative, while ketoconazole and orlistat were chosen as representative of neutral lipophilic drugs. Notably, ketoconazole was also selected as it can be seen as an in-between molecule, since it is a lipophilic compound, while possessing an ionizable imidazole ring which is present in known muscarinic ligands (e.g., pilocarpine). For the 6 selected compounds, muscarinic affinity studies were never reported apart from terfenadine which was investigated with the individual muscarinic subtypes showing a subtype selectivity for hM_3_ [23]. Though, the compound underwent biologic studies since its allosteric activity was never investigated.

### 2.3. Equilibrium and Kinetic Binding Studies

The affinity values of the six selected compounds for the five human muscarinic receptors subtypes (hM_1_–hM_5_) were determined by equilibrium binding experiments using [^3^H]-NMS as the radioligand. For easy comparison, W84 and gallamine were added as reference compounds endowed with a well-known allosteric activity on the hM_2_ subtype. Table 4 compiles the affinity values as expressed by pK_i_ parameters and reveals that 4 out of 6 tested compounds show an appreciable muscarinic affinity (pK_i_ greater than 4), a result which provides a truly encouraging validation for the adopted computational strategy. In detail, Table 4 shows that 3 out of 4 affinitive compounds belong to the first group of cationic ligands, a rather expected finding which confirms the mandatory nature of the ammonium head for interacting with the orthosteric binding pocket. The affinity of the fourth compound (ketoconazole) can be ascribed to the ion-pair stabilized by the charged imidazole ring thus further confirming the pivotal role of this interaction. In contrast and while being a basic molecule, aliskiren was found to be devoid of affinity for all muscarinic receptors. This result can be explained by considering both the size of this molecule and, in particular, the limited accessibility of the amino group which prevents a suitable interaction with Asp103 within the orthosteric pocket. As expected, molecules without positively ionizable groups are unable to occupy the orthosteric binding site.

A detailed analysis of the affinity values reveals that dequalinium is the sole ligand showing pK_i_ values greater than 7.0 with a weak selectivity for the hM_1_/hM_4_ receptors. In contrast to what was previously reported, terfenadine shows a non-negligible selectivity for hM_1_ with a difference of about one logarithmic unit with the other four muscarinic subtypes. Finally, ketoconazole and salmeterol show intermediate affinity values without discriminating between muscarinic subtypes. Collectively, terfenadine and ketoconazole show affinity values comparable with those of the reference compounds, while dequalinium reveals better values especially for the hM_1_ and hM_4_ subtypes.

In order to investigate possible interactions with the allosteric binding sites, the capacity of the selected compounds to affect the [^3^H]-NMS dissociation rate was evaluated using an one-point kinetic protocol focusing attention on the hM_2_ subtype only. Table 4 includes the so obtained log K_occ_ values, which encode for the reduction of the dissociation rate by the studied ligands. The reported values highlight the very remarkable log K_occ_ value for dequalinium, which is markedly higher compared to both gallamine and even W84, which is one of the most potent hitherto reported allosteric modulator for the hM_2_ subtype.

Even though the determined binding parameters do not allow a precise discrimination between orthosteric and allosteric interactions, the analysis of the pK_i_ and log K_occ_ values allows for some considerations. The truly notable log K_occ_ value of dequalinium, which in turn shows an intermediate pK_i_ value (on hM_2_), seems to be suggestive of a ligand which mostly act on the allosteric binding site similarly to what was observed for W84. The intermediate values of both pK_i_ and log K_occ_ of terfenadine can indicate the capacity to interact with both orthosteric and allosteric sites showing a possible bivalent profile. Finally, the low log K_occ_ values for ketoconazole and salmeterol combined with intermediate pK_i_ values can be indicative of ligands which mostly interact with the orthosteric binding cavity, even though also these two ligands possess a non-negligible capacity to reduce the [^3^H]-NMS dissociation rate. Aliskiren shows a very poor log K_occ_ value, thus suggesting that the inaccessibility of the protonated group affects the recognition by both orthosteric and allosteric sites. Finally, orlistat is completely inactive also on the allosteric site thus indicating that the polar contacts are also mandatory for a proper interaction within this and cannot be counterbalanced by extended hydrophobic contacts.

The discussed considerations are corroborated by docking analyses which allow an in-depth investigation of the binding sites with which the tested compounds preferentially interact. Figure 2 compares the best putative complexes as computed for dequalinium and ketoconazole. Figure 2B shows the key interactions stabilizing the complex with dequalinium and reveals that this ligand assumes a binding modes comparable to that of W84 (see Figure 2A). Compared to the W84 pose, the superior affinity of dequalinium can be ascribed to the charged quinolinium systems, which, along with the mentioned ionic interactions, can be engaged by mixed charge transfer plus π–π stacking interactions with a rich set of surrounding aromatic residues (such as Tyr80, Trp99, Tyr104, Tyr177, Tyr403, Tyr426 and Tyr430). As already seen for W84, a quinolinium ring partly occupies the orthosteric site without properly contacting Asp103.

Figure 2C shows the best putative complex as computed for ketoconazole and reveals that it is substantially accommodated within the orthosteric site where its charged imidazole ring contacts Asp103 and the 2,4-dichlorophenyl ring elicits π–π stacking interactions plus halogen bonds with surrounding aromatic residues (such as Tyr104, Tyr403, Tyr426 and Tyr430). The N-acetyl piperazine system is seen to approach key residues of the allosteric cavity but is unable to elicit clear interactions with them apart from weak H-bonds and a π–π stacking between the ligand’s amide function and Tyr177. Similar results are obtained by salmeterol and terfenadine which engage the orthosteric site stabilizing the key ion-pair with Asp103, while inserting within the allosteric pocket moieties unable to elicit significant contacts. Taken together, this first round of screening campaigns confirms the remarkable potential of bis-cationic molecules as allosteric ligands. The comparison of the docking results for dequalinium and W84 emphasizes the key role of the linker flexibility as well as of the delocalization of the positive charge on the aromatic systems, which boost the interaction pattern of dequalinium thus explaining its superior affinity parameters.

### 2.4. Second Targeted Screening Campaign

Based on the results of the previous campaign, a second targeted screening was carried out by paying attention on known marketed bis-cationic molecules. The selection was based on the previous docking simulations as well as on a conceptually similar study on MdfA ligands [24] in order to collect molecules able to explore the role of the linker length and of the arrangement of the positively charged moieties. As shown in Figure 1, such a selection identified three promising bis-cationic ligands (i.e., chlorhexidine, pentamidine and diminazene) to which a fourth very rigid molecule was added (paraquat) to better investigate the role of linker flexibility. Table 4 reports the affinity parameters also for the compounds of this second campaign. Chlorhexidine and pentamidine reveal an affinity profile comparable with that of gallamine showing pK_i_ values around 6 without significantly discriminating between individual subtypes, apart from a weak selectivity of chlorhexidine for hM_1_ and pentamidine for hM_2_. Diminazene shows intermediate pK_i_ values around 5, while paraquat appears to be a poor ligand with pK_i_ below 5. Both these last ligands do not discriminate between muscarinic subtypes. These results emphasize the crucial role of the linker length also for the recognition within the orthosteric cavity. When focusing on the hM_2_ subtype, affinity data confirms the key role of the arrangement of the positively charged moiety since the affinity increases when the ionized group has a terminal and accessible position (as seen in pentamidine) which facilitates its approach towards Asp103 (see below).

The linker length assumes an even more crucial role when considering the allosteric interactions since the two ligands endowed with longer linkers retain good log K_occ_ values (around 5) which are suggestive of a good capacity to affect the [^3^H]-NMS dissociation rate, while the two more rigid derivatives are substantially inactive (with log K_occ_ < 4). Collectively, the affinity parameters indicate that chlorhexidine can bind both orthosteric and allosteric binding sites with an overall profile superimposable to that of gallamine, pentamidine seems to prefer the orthosteric site, while diminazene and paraquat are modest orthosteric ligands.

**Figure 3:**
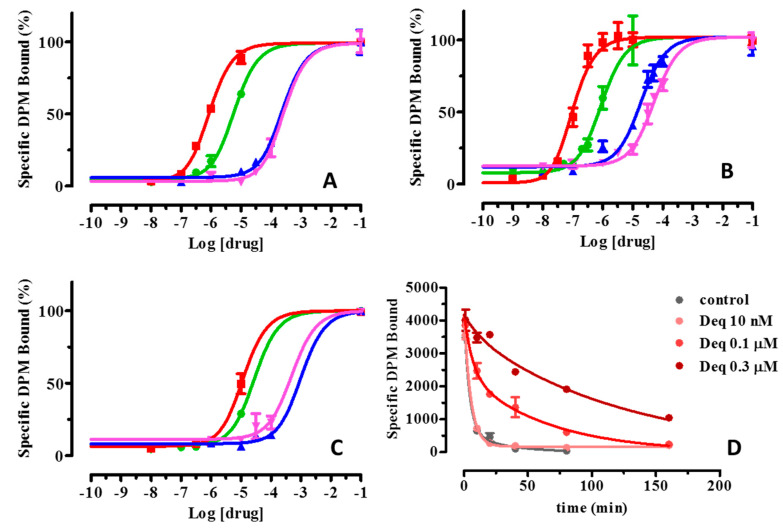
Effects on the [^3^H]-NMS dissociation rate of three most interesting retrieved compounds (dequalinium: red squares, chlorhexidine: blue tringles and pentamidine: pink tringles) plus the reference W84 compound (green circles) as derived by single time-off rate experiments on (**A**) hM_1_, (**B**) hM_2_ and (**C**) hM_5_. The data point at log [drug] = −1 represents the [^3^H]-NMS bound in the absence of added atropine and allosteric ligand; (**D**) shows a representative graph of the reduction of the dissociation rate (K_off_) and the corresponding t_1/2_ values of dissociation as obtained from full time course experiments for dequalinium on the hM_2_ subtype.

On these grounds, the capacity to reduce the [^3^H]-NMS dissociation rate of the three most interesting compounds (dequalinium, chlorhexidine and pentamidine) was also investigated on hM_1_ and hM_5_ subtypes. Table 5 and Figure 3A–C report the measured log K_occ_ and shows that all tested compounds possess a similar profile, which is also comparable with that of W84. In detail, the tested molecules show: (1) an overall selectivity towards hM_2_; (2) intermediate log K_occ_ values for hM_1_ and (3) very poor log K_occ_ values for hM_5_. This general trend confirms the greater structural diversity of hM_5_ compared to other muscarinic subtypes. While sharing the described general profile, dequalinium shows a very interesting allosteric activity also on hM_1_ subtype and retains a non-negligible allosteric effect even on hM_5_. The interesting allosteric activity of dequalinium on hM_1_ combined with its remarkable pK_i_ value (see Table 4) suggests that dequalinium can act as a bivalent ligand on the hM_1_ subtype.

Finally, and with a view to better investigating the notable allosteric effect of dequalinium on hM_2_ full time course experiments were performed at three different concentrations and during a monitored time of 160 min. As explained under Methods, these experiments allow the determination of the reduction of the dissociation rate (k_off_) and the corresponding t_1/2_ of dissociation. Figure 3D and Table 6 summarizes the full time course results and clearly confirms the dequalinium capacity to reduce the dissociation rate in a dose-dependent manner as evidenced by the t_1/2_ reduction of 4.8- and 17.3-fold at the concentration of 0.1 and 0.3 μM, respectively.

In order to better investigate the apparently bivalent profile of chlorhexidine which shows relevant and comparable pK_i_ and log K_occ_ values, Figure 2D depicts the corresponding computed complex and confirms that the ligand is conveniently inserted into both the orthosteric and allosteric cavities. In detail, the first biguanide function contacts Asp103 within the orthosteric cavity, where the 4-chloro phenyl ring stabilizes π–π stacking interactions plus a halogen bond with Asn404. Again, the second charged biguanide group is accommodated within the allosteric site where it stabilizes ion-pairs with Glu172 or Glu175 plus charge transfer interactions with Tyr177 and Tyr83. By contrast, pentamidine (complex not shown) is able to properly occupy the orthosteric cavity where the charged carboximidamide group contact Asp103, while being unable to stabilize ion-pairs within the allosteric cavity where the second charged phenyl carboximidamide moiety at most elicits π–π stacking with Tyr177 and Trp422.

## 3. Materials and Methods

### 3.1. Preliminary Virtual Screening Simulations

As mentioned in the Introduction, all performed docking simulations involved two resolved human hM_2_ structures: the inactive state bound to an antagonist (PDB Id: 3UON) [14] and the active state bound to the agonist iperoxo and the allosteric modulator LY2119620 (PDB Id: 4MQT) [15]. The downloaded structures were prepared as recently described [25]. Briefly, the structures were completed by adding hydrogens and the missed side-chains: to remain compatible to physiological pH, the residues Asp, Glu, Lys and Arg were considered ionized while Cys and His as neutral by default. The so completed protein structures were minimized by keeping the backbone atoms fixed to preserve the experimental folding. A set of 30 known allosteric ligands for hM_2_ was then collected from literature (see some reference compounds in Figure 1) [26,27]. Next, a purposely collected dataset of 2970 presumably inactive decoys was extracted from the ZINC database [28]. By considering that almost all chosen active ligands are characterized by a positive charge, the extracted decoys were selected so that their formal charge average was substantially superimposable to that of allosteric ligands (+1.71 vs. +1.67). Other physicochemical parameters considered in order to obtain an homogeneous dataset included molecular weight, lipophilicity and flexibility as encoded by the number of rotors. For all descriptors, the ranges covered by active and inactive compounds were roughly superimposable. The so obtained ligand database, in which the active compounds represent the 1%, was prepared for the following docking simulations by using automatic scripts implemented in the VEGA suite of programs as previously detailed [29]. Docking simulations were performed using PLANTS [30] and focusing the search within a 15 Å radius around the bound (and deleted) ligands to encompass both orthosteric and allosteric binding cavities in both hM_2_ structures. In detail and for each ligand, 10 poses were generated and ranked using the ChemPLP scoring function with speed equal to 1. Each computed pose was then optimized and rescored by ReScore+ [31] which computes the following scores: (1) ChemPLP, PLP and PLP95 as computed by PLANTS plus the corresponding normalized values; (2) XScore with its components; (3) APBS binding energies; (4) number of contacts and corresponding normalized values; (5) interaction energies as computed by VEGA including the hydrophobic interactions encoded for by the MLP Interaction score (MLPIns). By applying the binding space concept [22], each scoring function for a given molecule is defined by the following parameters (a) the best score value (as routinely done), (b) the score average and (c) the score range as derived considering all the ten generated poses. The calculated binding space parameters were finally used to develop consensus models by using the EFO approach [20]. In detail and for each analysis, 10 equations were developed including at most three variables by systematically combining all computed scores plus the corresponding binding space descriptors. After discarding interrelated and ineffective variables, the EFO approach exhaustively combines the input descriptors by means of a search algorithm which optimize a quality function based on both the early recognition (as encoded by EF 1%) and the distribution of active molecules within the entire ranking (as encoded by the skewness). The predictive power of these models was assessed by randomly subdividing the dataset into training (70%) and test (30%) sets and repeating this task 5 times.

### 3.2. Virtual Screening Simulations for Drug Repositioning

For drug repositioning, the DrugBank database was used [21]. This was filtered by removing molecules with less than 8 atoms or bigger than 1000 Da as well as molecules with counter-ions, metals or rare elements. In this way, a database including 5800 known molecules was collected and prepared as above described and underwent docking simulations on the best performing mAChR2 structures (as assessed by previous simulations, see below) by applying the same computational protocols previously described. Similarly, the computed poses were optimized and rescored by ReScore+ and the obtained scoring functions were used to rank the screened compounds based on the best predictive models as generated above. Based on these results, 6 promising compounds were selected also considering chemical diversity and availability.

### 3.3. Biologic Studies

All tested compounds were purchased from Merck KGaA (Darmstadt, Germany). The radioligand binding experiments were performed with membranes from Chinese Hamster Ovary (CHO) cells stably transfected with the five cloned human muscarinic receptor subtypes (hM_1-5_). Membrane fractions were prepared according to the protocol, which have been previously described [32]; membranes were stored at −80 °C until use. Nonspecific binding was defined by the radioactivity bound in the presence of 10 µM atropine.

#### 3.3.1. Equilibrium Binding Assays

The inhibition binding experiments were carried out at room temperature in polypropylene 96-well plates (Sarstedt, Verona, Italy) in a final volume of 250 μL of 25 mM Na/K phosphate buffer containing 5 mM MgCl_2_ at pH 7.4 (assay buffer), in the presence of 0.2 nM [^3^H]-N-methylscopolamine chloride ([^3^H]-NMS) (PerkinElmer Life and Analytical Science) and different concentrations of the tested compounds (0.001–100 µM). Aliquots of membranes (25–70 µg/mL) were added and incubated for 2 h at room temperature, filtered through UniFilter GF/B plates (PerkinElmer Life and Analytical Science, Monza, Italy) using a FilterMate cell harvester (PerkinElmer Life and Analytical Science, Monza, Italy). The filters were washed several times with ice-cold MilliQ water. Then, the plates were counted in a β-counter (TopCount NXT microplate scintillation counter, PerkinElmer Life and Analytical Science, Monza, Italy).

#### 3.3.2. Dissociation Kinetic Assay

##### *Full time* *course*

Dissociation binding assays were conducted in assay buffer at room temperature as above described [33]. Membranes were preincubated with 2 nM [^3^H]-NMS for 60 min at room temperature (mix membrane). Net dissociation of [^3^H]-NMS was initiated by the addition of 100 µL aliquots of this mixture to tubes which contained 1 µM atropine, with or without the indicated concentrations of test compound (final volume 1 mL): in this case atropine is used to prevent the reassociation of [^3^H]-NMS to the receptors. Nonspecific binding was measured in separately prepared tubes containing 100 µL of mix membrane plus atropine 10 µM.

After the appropriate time interval (0, 1, 10, 20, 40, 80 and 160 min), the dissociation was terminated by filtration through glass fiber filters grade MGB (Sartorius Italy S.r.l., Bagno a Ripoli, Firenze, Italy) that was soaked for 60 min in 0.05% polyethyleneimine, using a Brandell cell harvester (Biomedical Research and Development Laboratory, Inc. Atlas Drive, Gaithersburg, MD, USA); the filtration was immediately followed by three rinses with ice-cold MilliQ water. The filter-bound radioactivity was quantitated by liquid scintillation counting (Tricarb 1200TR, PerkinElmer Life and Analytical Science, Monza, Italy).

##### *One point* *kinetic assays*

Off-rate assays [34] were performed as described elsewhere [33] to estimate the affinity of the ligands for the [^3^H]-NMS-occupied receptor (log K_occ_). Briefly, a high concentration of membranes (about 0.1 mg/mL) was incubated with a high concentration of [^3^H]-NMS (2 nM) for 60 min at room temperature (mix membrane). Next, 100 µL aliquots were distributed into polypropylene tubes that were empty or in a final volume of 1 mL binding buffer with 1 µM atropine alone and in the presence of a range of concentrations of tested compound (1 nM–0.1 mM).

The effect on the radioligand dissociation of each tested ligand was determined at 0 min and at one time point, which was chosen to be ca 2.5 dissociation half-lives of [^3^H]-NMS alone from each subtype receptors. Later (80 min for hM_1_, 20 min for hM_2_ and 70 min for hM_5_), the samples were filtered and counted as previously reported. Non specific binding was measured as previously reported [33].

#### 3.3.3. Data Analysis

Data generated from binding assays were analyzed using Prism 5.02 (GraphPad Software, Inc., San Diego, CA, USA). Data points from radioligand inhibition binding curve were fitted to models using nonlinear regression equation to determine inhibitor potency (IC_50_) estimates, which were then converted to K_i_ values [35] as appropriate.

Data from one-point kinetic experiments were analyzed in order to obtain estimates of the affinity of an allosteric ligand for the [^3^H]-NMS occupied receptor (log K_occ_) in a single step, using an equation introduced in GraphPad Prism 5.02 [33,34].

Radioligand dissociation rates in absence or in presence of the allosteric modulator were analyzed by non linear regression according to the equation for mono-exponential decay using GraphPad Prism 5.02.

All the results are expressed as means ± SEM obtained from 3-4 independent experiments each one performed in duplicate.

## 4. Conclusions

As repeatedly reviewed, allosteric ligands can play key role in enhancing the subtype-selectivity of the orthosteric ligands and their relevance clearly parallels the difficulty in designing selective orthosteric ligands for a given biologic target [36]. The muscarinic receptors represent a clear example since they are implicated in several truly debilitating diseases (such as Alzheimer disease, schizophrenia and asthma), but the use of drugs variously acting on muscarinic receptors is woefully limited by the fact that even those molecules which reached the market are associated with several significant central and peripheral side effects. The unsuitable profile of these ligands is due to their poor selectivity by which they similarly bind all muscarinic receptors subtypes, a problem that can be ascribed to the very high degree of conservation among the key residues lying in the orthosteric cavity of the five muscarinic subtypes.

On these grounds, the great interest gained by muscarinic allosteric ligands in the last decades comes as no surprise when considering that the lower degree of conservation of residues lying in the allosteric cavity should permit the design of selective allosteric modulators [37]. The interest for the allosteric cavity is further increased by the possibility of developing selective bivalent ligands that are molecules able to occupy at the same time both orthosteric and allosteric pockets [38]. In the context of allosteric modulation, the hM_2_ receptor was extensively investigated and diverse allosteric ligands were proposed starting from ’80 when antidotes against organophosphate intoxication (such as W84 and its derivatives) were found to be able to exert allosteric activity on muscarinic receptors [39]. Since them, a significant number of novel allosteric modulators was reported in literature, even though their rational design was somewhat hampered by the lack of resolved structures for the muscarinic receptors [27].

Fortunately, the structures of the muscarinic receptors were resolved and now can be conveniently utilized in structure-based computational studies showing that even in “fields as well-ploughed as the muscarinic receptors” (cited by ref. [40]), they can add significant contributions by allowing the identification of new chemotypes or the repositioning of known molecules as reported here.

From a computational standpoint, the study emphasizes the potential of utilizing the binding space concept also in virtual screening contexts by simultaneously considering different reliable poses per ligand. Such a strategy appears to be particularly effective when simulating target structures, the binding cavity of which has a non-optimized architecture (as seen for studies based on 3UON structure) but can enhance the performances also of optimized target structures by accounting for the often neglected entropic factors.

In summary, the present study describes a targeted computational protocol which allowed the identification of ligands acting on muscarinic receptors with different mechanisms. In particular, the reported VS campaign led to the identification of a very potent allosteric modulator which can represent the starting point to design new ligands with both pure allosteric and bivalent profiles. In addition, the study revealed the promising profiles of terfenadine, chlorhexidine and pentamidine on the muscarinic receptors and suggested that ketoconazole can be considered as an interesting scaffold to design novel imidazole-based muscarinic ligands.

## Figures and Tables

**Table 1:** Predictive performances of the generated models based on the performed virtual screening simulations as encoded by the highest and the average Enrichment Factor (EF) 1% values.

Utilized Protein(s) (PDB Id)	Scores of Best Pose Only		Score Averages of 10 Poses		Score Averages and Ranges of 10 Poses	
	EF 1% Mean ^a^	Best EF 1%	EF 1% Mean ^a^	Best EF 1%	EF 1% Mean ^a^	Best EF 1%
3UON	18.1	24.1	42.8	48.2	47.5	51.6
4MQT	59.4	65.4	54.4	65.4	64.2	68.8
3UON + 4MQT ^b^	60.7	65.4	59.0	65.4	66.1	68.8

^a^ Mean values as computed by averaging the EF 1% values of the 10 best consensus equations generated by the EFO method; (b) 3UON+4MQT indicates the performances of the consensus models generated by combining the scores as computed for the two simulated hM_2_ structures.

**Table 2:** Best enrichment factor optimization algorithm (EFO)-based equations as developed by docking simulations on the two considered hM2 structures. Equations based on the combination of the two proteins were omitted because they were identical to those produced by 4MQT only.

Utilized Protein (PDB Id)	Score Types	Equation	EF 1%
3UON	Best scores	1.00 Contacts_NORM_HEVATMS__Best + 0.031 ChemPLP_Best − 0.014 PLP_Best ^a^	24.09
3UON	Mean scores	1.00 ChemPLP_Mean − 0.75 PLP_Mean − 3.42 PLP95_NORM_HEVATMS__Mean	48.18
3UON	Means + ranges	1.00 MLP_INS__Range + 0.08847263 ChemPLP_Mean − 1.51 PLP95_NORM_HEVATMS__Mean	51.62
4MQT	Best scores	1.00 ChemPLP_NORM_HEVATMS__Best + 0.0073 PLP_Best − 4.00 PLP95_NORM_HEVATMS__Best	65.39
4MQT	Mean scores	1.00 ChemPLP_NORM_HEVATMS__Mean − 4.13 PLP95_NORM_HEVATMS__Mean + 1.60 XScore_HM_Mean	65.39
4MQT	Means + ranges	1.00 Contacts_NORM_WEIGHT__Range + 0.37 ChemPLP_NORM_WEIGHT__Mean − 0.030 PLP95_NORM_HEVATMS__Mean	68.83

^a^ For sake of clarity, the suffixes best, mean and range refer to best score value, score average and score range, respectively. Similarly, the subscripts NORM_HEVATMS and NORM_WEIGHT stand for normalized score values per the number of heavy atoms and weight, respectively.

**Table 3:** Top ranked 30 marketed drugs among which 6 promising ligands (in bold) were selected and tested. LogP values and bioactivities were taken from DrugBank [16].

Compound	Charge	logP	Known Bioactivity
adefovir dipivoxil	0	1.5	reverse transcriptase inhibitor
**aliskiren**	**1**	**3.3**	**renin inhibitor**
almitrine	1	4.1	Na/K-transporting ATPase subunit alpha-1 agonist
ambenonium	2	2.3	cholinesterase inhibitor
bepridil	1	5.2	calcium channel blocker
bimatoprost	0	3.4	structural analogs of prostaglandin
carvedilol	1	3.1	beta adrenoceptor blocker
cetirizine	0	2.8	histamine H1 antagonist
deferoxamine	1	0.9	chelating agent
demecarium	2	0.6	cholinesterase inhibitor
**dequalinium**	**2**	**0.2**	**antiseptic and disinfectant agent**
dinoprostone	−1	2.8	naturally occurring prostaglandin derivative
fexofenadine	1	5.0	histamine H1 antagonist
hexafluronium	2	1.8	neuromuscular blocking agent
iloprost	−1	4.2	synthetic analog of prostacyclin
**ketoconazole**	**0**	**4.4**	**imidazole antifungal agent**
lapatinib	1	5.2	tyrosine kinases inhibitor
latanoprost	0	4.2	prodrug analog of prostaglandin
mupirocin	−1	2.2	antibacterial agent
**orlistat**	**0**	**7.5**	**pancreatic lipase inhibitor**
oxybutynin	1	4.3	antimuscarinic agent
phytonadione	0	9.3	vitamin K1
pimozide	1	6.3	antipsychotic agent
**salmeterol**	**1**	**3.8**	**beta2-adrenergic receptor agonist**
silodosin	1	3.0	α1-adrenoceptor antagonist
terconazole	0	4.5	imidazole antifungal agent
**terfenadine**	**1**	**5.9**	**histamine H1 antagonist**
travoprost	0	4.6	synthetic prostaglandin analog
vilazodone	1	4.2	serotoninergic agent
ximelagatran	1	1.4	Anticoagulant agent

**Table 4:** Inhibition binding constants, pK_i_, describing estimated equilibrium binding affinity for human cloned muscarinic receptors plus the affinity estimates (log K_occ_) at the [^3^H]-NMS-occupied muscarinic hM_2_ subtype. Values are reported as means of 3-4 experiments ± SEM.

Compound	pK_i_ hM_1_	pK_i_ hM_2_	pK_i_ hM_3_	pK_i_ hM_4_	pK_i_ hM_5_	Log K_occ_ hM_2_
**Reference Ligands**						
W84	6.05 ± 0.43	5.21 ± 0.15	5.26 ± 0.25	5.30 ± 0.13	5.04 ± 0.16	6.54 ± 0.13
gallamine	6.38 ± 0.16	6.91 ± 0.10	5.69 ± 0.33	6.10 ± 0.12	5.66 ± 0.06	5.19 ± 0.06
**First round**						
dequalinium	7.38 ± 0.32	6.18 ± 0.16	6.77 ± 0.27	7.16 ± 0.14	6.80 ± 0.10	7.72 ± 0.26
terfenadine	6.11 ± 0.14	4.8 ± 0.04	5.12 ± 0.21	4.96 ± 0.15	5.19 ± 0.20	4.74 ± 0.06
ketoconazole	5.43 ± 0.04	5.12 ± 0.17	5.01 ± 0.12	5.34 ± 0.10	5.1 ± 0.23	4.21 ± 0.08
salmeterol	5.49 ± 0.01	4.98 ± 0.13	4.92 ± 0.2	5.01 ± 0.12	4.9 ± 0.14	4.06 ± 0.07
aliskiren	<4	<4	<4	<4	<4	3.60 ± 0.10
orlistat	<4	<4	<4	<4	<4	<3
**Second Round**						
chlorhexidine	6.30 ± 0.10	5.42 ± 0.27	5.67 ± 0.04	5.79 ± 0.06	5.65 ± 0.05	5.24 ± 0.02
pentamidine	5.60 ± 0.11	6.04 ± 0.08	5.67 ± 0.12	5.88 ± 0.03	5.76 ± 0.05	4.73 ± 0.06
diminazene	5.5 ± 0.18	5.25 ± 0.12	5.32 ± 0.08	5.05 ± 0.05	5.32 ± 0.10	3.81 ± 0.28
paraquat	4.43 ± 0.09	4.63 ± 0.13	4.70 ± 0.07	<4	4.83 ± 0.13	3.12 + 0.31

**Table 5:** Affinity estimates (log K_occ_) at the [^3^H]-NMS-occupied muscarinic hM_1_, hM_2_ and hM_5_ subtypes plus the corresponding selectivity ratios. Values reported as means of 3–4 experiments ± SEM.

Compound	hM_2_	hM_1_	hM_5_	hM_2_/hM_1_	hM_2_/hM_5_
W84	6.46 ± 0.09	5.79 ± 0.09	4.74 ± 0.27	4.6	52.5
Dequalinium	7.72 ± 0.26	6.69 ± 0.10	5.48 ± 0.16	10.7	174
Chlorhexidine	5.24 ± 0.02	4.09 ± 0.11	3.73 ± 0.17	14	32.4
Pentamidine	4.73 ± 0.06	4.18 ± 0.15	3.74 ± 0.19	3.5	9.8

**Table 6:** Reduction of the dissociation rate (K_off_) and the corresponding t_1/2_ values of dissociation as obtained from full time course experiments at three different concentrations and during a monitored time of 160 min.

	K_off_ (min^−1^) (±SEM)	t_1/2_ (min) (95% C.I.)
Control (NMS)	0.16 ± 0.02	4.41 (3.53–5.88)
Dequalinium 1 nM	0.15 ± 0.02	4.51 (3.62–5.99)
Dequalinium 0.1 µM	0.033 ± 0.003	21.02 (17.95–25.37)
Dequalinium 0.3 µM	0.009 ± 0.001	76.54 (64.83–93.42)

## Abbreviations

EF	Enrichment factor
EFO	Enrichment factor optimization
GPCR	G protein-coupled receptor
hM	Human muscarinic acetylcholine receptor
hmAChR	Human muscarinic acetylcholine receptor
NMS	N-methyl scopolamine
QNB	Quinuclidinyl benzilate
VS	Virtual screening
